# Development and Evaluation of Sodium Alginate/Carbopol 934P-Co-Poly (Methacrylate) Hydrogels for Localized Drug Delivery

**DOI:** 10.3390/polym15020311

**Published:** 2023-01-07

**Authors:** Ayesha Mahmood, Asif Mahmood, Mohamed A. Ibrahim, Zahid Hussain, Muhammad Umar Ashraf, Mounir M. Salem-Bekhit, Ibrahim Elbagory

**Affiliations:** 1 Faculty of Pharmacy, University of Lahore, Lahore 5400, Pakistan; 2 Department of Pharmacy, University of Chakwal, Chakwal 48800, Pakistan; 3 Department of Pharmaceutics, College of Pharmacy, King Saud University, Riyadh 11451, Saudi Arabia; 4 Department of Pharmaceutics and Pharmaceutical Technology, College of Pharmacy, University of Sharjah, Sharjah 27272, United Arab Emirates; 5 College of Pharmacy, Northern Border University, Arar 1321, Saudi Arabia

**Keywords:** diloxanide furoate, sodium alginate, Carbopol 934P, localized delivery, free radical polymerization

## Abstract

This research was carried out to create a pH-responsive polymeric system for the targeted drug delivery of Diloxanide furoate. It relied on sodium alginate (Na-Alg) and Carbopol 934P as building blocks. Using an aqueous free radical polymerization method, SCH1-SCH12 was created with varying polymer, MAA, and MBA input ratios. Positive outcomes were seen in the swelling and release profiles at higher pH levels. Hydrogel formation, as well as component compatibility, thermal stability, and Diloxanide furoate loading, were all validated by instrumental characterization. A drug loading percentage of 83.56% was determined, with the swelling reaching 743.19%. For the formulation with MBA, the gel fraction was 94.58%. The release of diloxanide furoate increased to 91.77% at neutral pH. The formulation containing Carbopol 934P provided the highest mucoadhesion force (3993.42 dynes/cm^2^). The created hydrogel has been shown to be biocompatible by toxicological testing of the network. Based on the findings, the created polymeric nexus proved promising for pH-dependent localized and regulated delivery of Diloxanide furoate.

## 1. Introduction

Over the last several years, controlled localized drug delivery carriers have supplanted more traditional delivery carriers, leading to more widespread treatments. This curiosity stems from the clinical requirement of precisely dosing controlled drugs to specific biological regions for maximum pharmacological effect. Novel biodegradable polymers have made a localized distribution of medication to particular areas a viable method for lowering systemic toxicity [1]. Over the last several decades, the use of colonic localization for treating disorders such as inflammatory bowel or Crohn’s disease, irritable bowel syndrome [IBS], colon or colorectal cancer, and amebiasis has received widespread support. In addition to being an important location for absorption, the colon has several other appealing characteristics. These include a less hostile environment, neutral pH, a longer transit time, a high sensitivity to absorption enhancers, and several enzymes for polysaccharides.

Using polymers like pectin, dextran, and Na-Alg is a promising strategy for intestinal-specific delivery [Na-Alg). Enzymes in the digestive tract break those down. Hydrogels, made of polymer networks, show promise as controlled delivery methods. Hydrogels, three-dimensional polymeric networks, may be made from either synthetic or natural substances. They can be shaped easily because of their flexibility and high-water content. Hydrogels formed from these polymers have outstanding mucoadhesive properties due to the pendant groups’ adhesion to mucous membranes and the polymers’ pH responsiveness thanks to the pendant groups’ acidic or basic nature, allowing for tailored distribution. The stomach’s pH causes it to contract when the pH is low and expand when the pH high, like in the intestine. Because of their superior control over drug release, hydrogels have become an attractive approach for administering medications without unwanted side effects [2]. According to research by Liang et al., the effectiveness of treating local infections depends on how long localized drug delivery carriers may persist at the absorption site. The researchers created chitosan grafted onto hydrocaffeic acid and oxidized pullulan so that amoxicillin could be administered selectively [3].

Various “conventional” chemical techniques may be used to create hydrogels. There are both one-step methods, such as polymerization and the parallel cross-linking of multifunctional monomers, and multi-step methods. The advancements in polymer science have allowed the synthesis of polymeric networks to be controlled on the molecular level, allowing for the design of networks with specific properties. Cross-linking density, biodegradability, mechanical strength, and other aspects of biological and chemical responses to stimuli may be altered by using various polymers, monomers, etc., and by using alternative methods [4].

Na-Alg may be found as a white to yellowish fibrous or granular powder with almost no discernible odor. It dissolves easily in water but not in chloroform, ether, or alcohol. It leads to the formation of very thick colloidal solutions in water [5]. Na-Alg changes into a porous alginic acid skin in an acidic environment while changing into a soluble layer in the colon [6]. This phenomenon might be used to tailor the release profile, although the fast breakdown of Na-Alg at high pH results in burst release [7].

Carbopol 934P is a synthetic hydrophilic biocompatible, biodegradable stable polymer that increases the delivery system’s toughness and flexibility. The cosmetic and pharmaceutical sectors have taken advantage of its excellent swelling and thickening characteristics. Regarding release patterns, hydrophilicity and the presence of -COOH groups are crucial since they impart equilibrium swelling [8]. Such polymers show great promise for being used as medicinal carriers, especially in controlled-release formulations.

Acrylates, such as acrylic acid, hydroxyethyl methacrylate, methacrylic acid, and others, may be included in the formulation of a carrier to make it pH-responsive. Methacrylic acid-based networks are sensitive to their surroundings. Because its carboxylic groups are ionized into carboxylate ions at the basic pH of GIT, MAA is crucial, as it modulates swelling behavior [9].

Diloxanide furoate (DF) is phenyl 4-(2,2-dichloro-*N*-methylacetamido) furan-2-carboxylate, and it is a part of the biopharmaceutical classification system (BCS) class II. Solubility is low in ether, water, and alcohol but high in chloroform [10]. This amebicide is either used alone to treat non-dysenteric amoebiasis or in conjunction with other amebicides that operate on the tissue layer of the intestine (metronidazole or Tinidazole). A dosage of 500 mg three times a day for ten days is efficacious as monotherapy or in conjunction with any tissue amebicide, including metronidazole and Tinidazole [11]. Hydrolysis shortens its half-life in live organisms to only 3 h. The kidneys are responsible for 90% of the removal process after the medication has been converted to its glucuronide form; the liver is responsible for the remaining 10% [12].

Hydrogels based on Carbopol 934P and Na-Alg were created and studied for the localized and controlled delivery of DF in the gut to treat amebiasis, to avoid the burst release inherent with preparations of Na-Alg alone at higher pH, and to modulate the drug release pattern.

## 2. Materials

Thankfully, diloxanide furoate was donated by Adamjee Pharmaceuticals (DF). The St. Louis, MO, USA headquarters of Sigma-Aldrich Co., was the source of our materials. Na-Alg, Carbopol 934P, 99.9% Methacrylic acid (MAA), benzyl peroxide, 99.9% *N*,*N*-methylene bisacrylamide (MBA), and 99.7% methanol were among the materials used. For the acquisition of NaOH, KH_2_PO_4_, NaH_2_PO_4_, and NaCl, we reached out to Dae-Jung Chemical Company in Gyeonggi-do, Korea. All chemicals used were of a pure and undamaged analytical grade. The distilled water was just made in a research facility at the University of Lahore’s Faculty of Pharmacy.

### 2.1. Na-Alg and Carbopol 934P (a Co-Polymer of Methyl Methacrylate) Hydrogels

By altering the polymer ratios to the monomer to cross-linker, we created twelve distinct formulations (SCH-1 to SCH-12) using the free radical polymerization approach (Table 1). A measured amount of Carbopol 934P (CP) was added to the water at 40 degrees Celsius. The mixture was stirred at a continuous rate of 250 revolutions per minute (rpm) until a clear solution formed. At room temperature, Na-Alg was dissolved in water with constant stirring. A homogeneous polymer mixture was achieved by adding CP to the Na-Alg solution. Solubilized benzoyl peroxide (BPO) was added to the polymeric mix, followed by the monomer solution of MAA. They stirred the reaction mixture for an additional five minutes. Lastly, the cross-linker MBA was added to the reaction mixture while continuously stirring.

Then, the mixture was sonicated for 5–10 min to eliminate any remaining oxygen and air bubbles. Right away, the concoction was poured into test tubes. Aluminum foil-wrapped glass vials were submerged in a 45°C water bath. The first hour saw a hold at this temperature, followed by 50 degrees Celsius for 2 hours, 55 degrees for 2 hours, 60 degrees for 16 h, and 65 degrees for 2hours. After 24 h, the test tubes were removed from the water bath, brought to room temperature, and the hydrogel cylinders were removed by fracturing the test tubes with the wooden back of the test tube holder. The hydrogel cylinders were washed for several minutes under running water to flush out any glass splinters that may have made their way inside. The hydrogel was rolled out, and then cut into 8 mm discs and cleaned three times in a 70:30 ethanol: water solution to remove any residue or unreacted monomer. The discs were air-dried at room temperature before being heated to 45 degrees Celsius in a hot air oven (Memmert, Tokyo, Japan).

**Figure 1:**
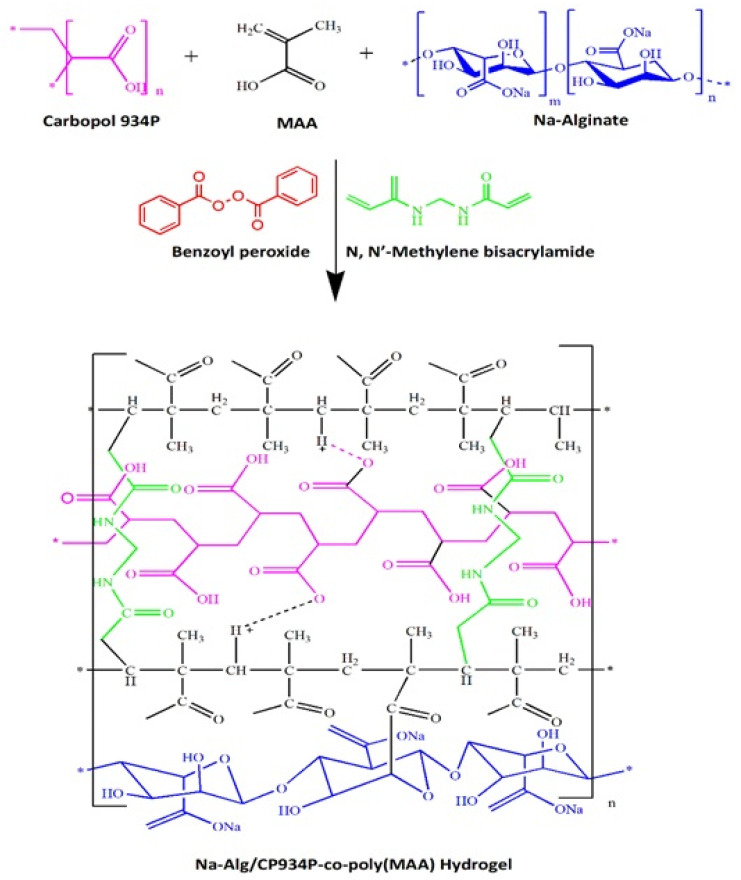
Proposed chemical structure of Na-Alg/CP-co-poly(methacrylate) hydrogel.

The cross-linking of carbopol 934P with the terminal carbon of cross-linker MBA produced a sandwich structure. Also, the oxygen atom of methacrylic acid (MAA) was hydrogen-bonded by the hydrogen atom of Carbopol 934P, creating an electrostatic link between the two molecules. The sodium alginate was able to form a connection with the MAA after having a water molecule removed. The potential chemical bonding and electrostatic interactions in the synthesized Na-Alg/CP-co-poly (methacrylate) hydrogel are further elaborated in Figure 1.

### 2.2. Diloxanide Furoate Loading (%)

DF was introduced into the hydrogel discs by swelling-assisted diffusion. Before immersing the dried hydrogel discs in a 1% (*w*/*v*) DF solution, we weighed them precisely on a digital scale (AUW 220, Shimadzu, Tokyo, Japan). The DF solution comprised 70% methanol and 30% phosphate buffer (pH = 7.4). When the discs were submerged in water, we let them swell until they reached a constant diameter. After being soaked in the drug solution, the discs were removed and washed quickly in water to remove any remaining drug molecules. Before being heated at 45 degrees Celsius, the discs were allowed to air dry. The drug-loaded hydrogel’s mass was determined as follows:

(1) $$Drug\;Loading\;\left(\%\right)=\frac{W_{D}-W_{d}}{W_{d}}\times 100$$

*W_D_* = Hydrogel disc with DF loaded, at final weight; *W_d_* is the initial weight of hydrogel discs before loading [13].

### 2.3. Equilibrium Swelling Studies

Swelling tests were used to evaluate the pH-responsive behavior of the produced hydrogels. The experiments employed buffer solutions at 37 °C, with pH levels of 1.2 and 7.4. Hydrogel discs, already weighed, were put into 1.2 and 7.4 pH buffer solutions independently. The discs were taken out of the solutions 2, 4, 6, 8, 12, 16, 20, 24, 28, 32, and 36 h after being immersed. Each time a disc was taken out, it was wiped down with blotting paper to remove any remaining adherent water and reweighed. We kept tracking the 8gains in weight until we reached a point when we were no longer in a state of balance. The equilibrium swelling (in%) for each formulation was determined using Equation (2) [14].

(2) $$Equillibrium\;Swelling\;\left(\%\right)=\frac{W_{s}-W_{d}}{W_{d}}\times 100$$

*W_s_*: the swollen disc’s weight at regular intervals; *W_d_*: the initial dried disc’s weight.

### 2.4. Sol-Gel Fraction Analysis

Sol-gel analysis was used to calculate the number of unreacted components in the hydrogel network. Hydrogel discs were fabricated and then divided into pieces with a diameter of between 2 and 4 mm, after which they were dried at ambient temperature and then in an oven at 45 °C until a constant weight was attained (W0). The dried materials were boiled for four hours in the Soxhlet apparatus to remove the compounds of interest. After 4 h, we dismantled the machine, transferred the pieces to a glass petri dish, and let them air dry for 24 h before drying them at 45 °C [15]. We determined the Sol-gel percentage by reweighing the samples after drying.

(3) $$Sol\;Fratcion\;\left(\%\right)=\frac{W_{0}-W_{i}}{W_{0}}\times 100$$

(4) $$Gel\;Fraction\;\left(\%\right)=100-Sol\;fraction$$

The dry gel’s weight before and after boiling is denoted as *W*_0_ and *W_i_*, respectively.

### 2.5. FTIR Spectroscopy

Hydrogel network structural cross-linking and component compatibility were analyzed using Fourier-transformed infrared spectroscopy (FTIR). The spectra of pure DF, Na-Alg, carbopol-934P, MAA, and the produced hydrogel were recorded using an FTIR spectrophotometer (Thermos Fischer Scientific Nicolet, 6700TM, Waltham, MA, USA). Crushed samples were combined with 12 mm pellets of KBr (Merck IR spectroscopy grade) to acquire spectra in the 4000–400 cm^−1^ range [16].

### 2.6. DSC/TGA Analysis

The DSC and TGA measurements verified the glass transition temperatures and the thermal stability of the created network compared to pure components at elevated temperatures. Differential Scanning Calorimetry (DSC) analysis was conducted using TA instruments (Model: DSC 2910, TA Instruments Inc., New Castle, DE, USA). Two- to five-milligram samples were heated in an oven at temperatures between zero and four hundred degrees Celsius. During heating, 20 mL/min of nitrogen flow and 20°C/min of heat were employed [13].

The materials were analyzed using a thermogravimetric analyzer (TGA system; Q600 TA V8.3 Build 101 Thermal Analysis system; TA instruments). After passing through sieve no. 40, the hydrogel samples (0.5–5 mg) were transferred to an aluminum pan, an open microbalance that is dynamically heated from 0 to 500 degrees Celsius at a rate of 20 degrees per minute in a nitrogen environment [14].

### 2.7. X-ray Diffraction (XRD) Studies

X-ray diffraction analysis was used to establish whether the medication, polymer, and network were crystalline or amorphous. The analysis was performed at room temperature using an X-ray diffractometer (Bruker; Karlsruhe, Germany). Scanning a range of 2 from 10°–70°, peaks were obtained while processing the samples using copper Kα as the radiation source [17].

### 2.8. Energy Dispersive Spectroscopy

To ascertain the elemental composition and loading of DF, the EDX spectra of the developed unloaded and DF-loaded networks were recorded using an EDX spectrophotometer (INCA 200 m oxford, Abingdon, UK) [18].

### 2.9. SEM Analysis

The generated hydrogel’s surface morphology was studied using scanning electron microscopy (SEM). To dry the chosen discs under a vacuum, they were placed on metal stubs and secured with double-sided adhesive tape before being placed in the vacuum chamber. A sputter coater was used to apply a layer of gold 10 mm thick, which was then examined at several magnifications [19].

### 2.10. In-Vitro Drug Release and Kinetic Modeling of Release Data

Using the USP dissolving equipment II and buffers with pH 1.2 and 7.4, an in vitro drug release investigation of the DF-loaded polymeric networks was performed. The discs were submerged in 900 cc of dissolving media, and the paddle speed was maintained at 50 rpm throughout the process. Throughout the experiment, the medium was kept at a constant 37 ± 2 °C. At 0.5, 1, 2, 4, 6, 8, 10, 12, 16, 20, 24, 36, 48, and 72 h, aliquots of 5 mL were taken from the vessel’s center and replaced with an equal amount of newly produced buffer to keep the medium at constant sink conditions. After filtration, the samples were analyzed using a UV Visible Spectrophotometer set to a maximum wavelength of 259.8 nm [20].

The optimal model and mechanism of DF release from the designed hydrogels were identified by processing the dissolution data using a DD solver add-in, Microsoft Excel-based application. We accomplished this by considering various mathematical kinetic models, such as the First Order (FO), Zero Order (ZO), Higuchi (Hig), and Korsmeyer-Pappas (KP) models. The n value was utilized to evaluate the drug release pattern, while the R^2^ value was used to choose the most appropriate model. If n is less than 0.45, the transport is characterized by Fickian diffusion; if it is between 0.45 and 0.89, the result is non-Fickian or anomalous transport; and if it is more than 0.85, then the result is super case II transport. The following are the equations used in these models:

(5) $$\mathrm{ZO}\;\;\;F_{t}=F_{o}-K_{o\;}t$$

(6) $$\mathrm{FO}\;\;\;lnln\;\left(1-F\right)=K_{1}\;t$$

(7) $$\mathrm{Hig}\;\;\;F_{t}=K_{H}t^{1/2}$$

*F*: the released drug fraction of *DF* in the hydrogel at time t (0 for initial amount fraction); *K_o_*, *K*_1_, and *K_H_* are ZO, FO, and Hig models rate constants, respectively.

(8) $$\mathrm{KP}\;\;\;\frac{Mt}{M^{\propto }}=k_{kp}t^{n}$$

*Mt/M∞* corresponds to the amount of DF released at time *t*, *K_kp_* to the rate constant, and n to the release exponent [21].

### 2.11. Experimental Mucoadhesion Ex-Vivo

**Figure 2:**
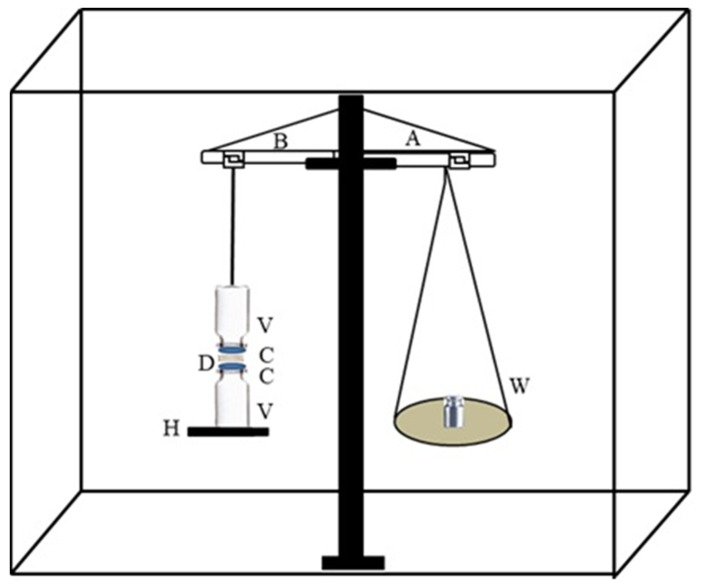
Mucoadhesive force measurement apparatus. A is the modified beam balance, B is the arm attached to the vial, V is glass vials, C is mucosal membranes, H is the height adjustment pan, W is the weights used to detach vials, and D is the hydrogel disc.

Experiments on the Ex-vivo Mucoadhesion of Na-Alg/CP-co-poly(methacrylate) hydrogels were conducted utilizing modified beam balance equipment, as illustrated in Figure 2.

The intestinal tissue of a newly slaughtered rabbit was dissected, turned upside down, and washed in a NaCl solution of 0.9% before being placed in phosphate buffer saline at 37 degrees Celsius for half an hour. The intestinal mucosa receptors were two V-shaped metal caps placed on glass vials. The intestinal mucosal membranes (4 cm in diameter) were glued to the outside of the vials using cyanoacrylate adhesive. The height adjustment pan (H) and the balance were attached to the first vial, while the second was connected to the third vial (B). The left pan of the beam has counterweights hung from it for balance (W). Hydrogel discs (D) were positioned between the mucosal tissues of the two vials (shown in Figure 2) by putting them on a height adjustment pan and then lowering the top vial.

The vials were eventually able to be removed due to the steadily increasing weights. The formula used to determine the detachment force as a measure of mucoadhesion was as follows:

(9) $$Detachment\;stress\;\left(dynes/cm^{2}\right)=m\times g/A$$

where *m* is the mass in grams needed to separate two mucosal membranes, *g* is the acceleration due to gravity, and *A* is the area of tissue removed before attachment.

### 2.12. Toxicity Evaluation

The Organization for Economic Co-operation and Development (OECD)’s guidelines for acute oral toxicity testing were used to evaluate the synthesized hydrogels. The research procedures were reviewed by the Institutional Research Ethics Committee (IREC) in the School of Pharmacy at the University of Lahore. It approved the proposal in a letter dated 2020–02-28, including the notification number IREC-2020-28. A week was sufficient to acclimatize 12 healthy albino rabbits since there was a 12-h day/night cycle, enough food and water, and an ambient temperature of 22–30°C. Each of the two groups of rabbits consisted of six animals. They constituted the treatment and control groups created out of the sample pool. The control group had access to unlimited food and drink. The treatment group rabbits were given 2–4 g of powdered hydrogel formulation per kilogram of body weight after 12 h of fasting and free access to water. The rabbits in both groups were physically examined for a number of traits on day 14. At 1, 7, and 14 days, blood samples were taken to check several biochemical indicators. These included the complete blood count, liver function, kidney function, and lipid profile. On day 14, similar to the control group, all rabbits in the therapy group had their vital organs removed for pathophysiological study [17,22].

## 3. Results and Discussion

### 3.1. Drug Loading (%)

**Figure 3:**
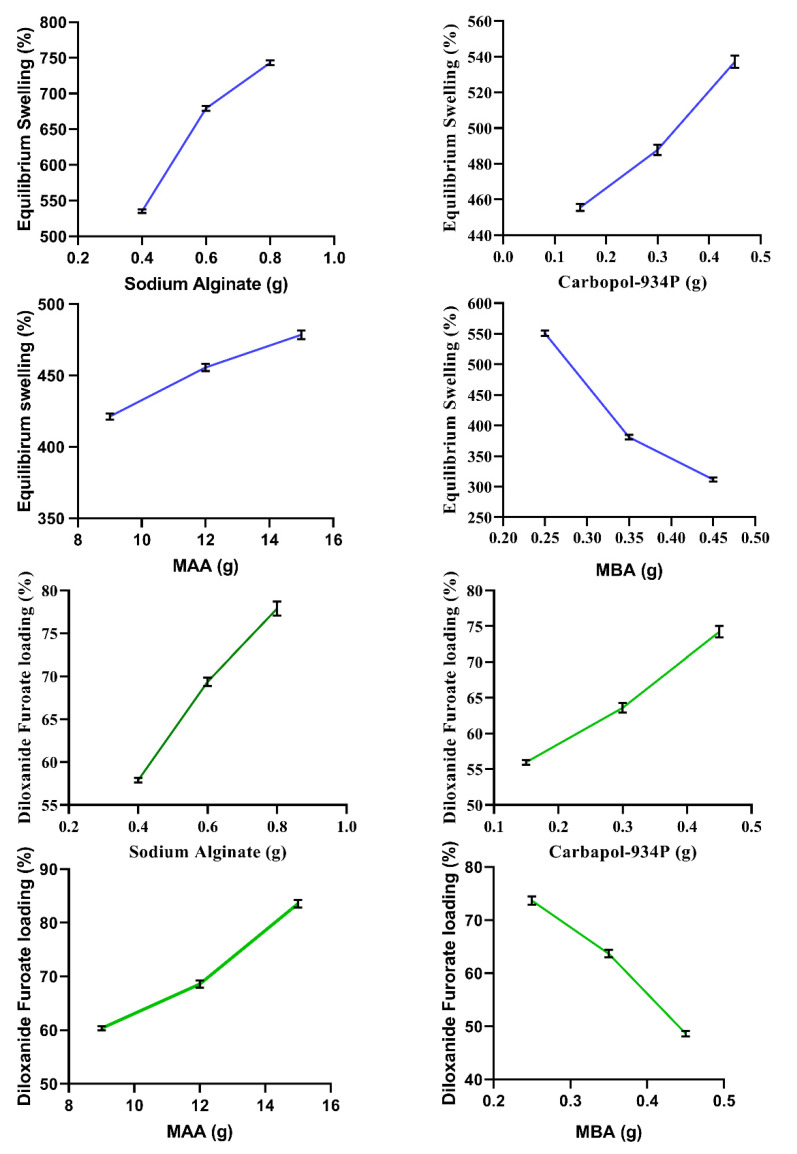
Effect of Na-Alg, CP-934P, MAA, and MBA on the equilibrium swelling (%) and DF loading (%) into developed hydrogels.

The feed content ratio significantly impacts DF loading into the developed polymeric network. Drug loading (%) is associated with the swelling behavior of the polymeric network. Higher Na-Alg, CP-934P, and MAA contents resulted in higher loading capacities of the polymeric networks. DF loading (%) increased from 57% to 77% in formulations (SCH-1 to SCH-3) with variable Na-Alg contents, which was attributed to the low cross-linking tendencies inherent in the Na-Alg structure, which causes high swelling capacities and hence more significant drug loading. Also, at higher MAA and Na-Alg contents, the resulting viscosity and density can retain a higher drug load in the hydrogel [23]. An increasing trend of DF loading from 55% to 74.21% (SCH-4 to SCH-6) and from 60.36% to 83.56% (SCH-7 to SCH-9) was also recorded after increasing the CP-934P and MAA contents, respectively. At higher MAA and Na-Alg contents, the viscosity and density of the hydrogel system were higher, resulting in the retention of higher drug loads within the developed network [23]. The increased drug loading (%) at a higher Carbopol -934P content is associated with improved swelling secondary to electrostatic repulsion among the pendant anionic groups [24]. DF loading decreased from 73.69% to 48.66% (SCH-10 to SCH-12) when the MBA contents were increased (Figure 3). This may be attributed to less swelling due to a higher cross-linking density and higher content of MBA.

### 3.2. Equilibrium Swelling (%)

Swelling studies evaluated the pH-responsive behavior of the developed hydrogel. Swelling studies revealed that constituents and feed ratios influence the hydrogels’ swelling behavior. All 12 formulations (SCH-1 to SCH-12) were analyzed for their pH-dependent behavior by placing them in buffers of 1.2 and 7.4 pH. At pH 1.2, all formulations exhibited minor swelling, whereas, at pH 7.4, significant and variable swelling (%) was demonstrated by all formulations.

### 3.3. Effect of Carbopol 934P on Swelling

After increasing the content of Carbopol-934 P in the hydrogel (SCH-1 to SCH-3), an enhancement of the swelling from 455.55% to 537.26% was noted at basic pH of 7.4. Improved swelling at a higher CP-934P content may be ascribed to the high degree of ionization of the carboxylic groups of the CP at basic pH. The ionized anionic –COO^−^ groups promote electrostatic repulsion, due to which a reduction of the H-bond linkages within the chains occurs, resulting in the expansion of the polymeric chains and an influx of swelling media, and hence enhanced equilibrium swelling resulted.

### 3.4. Effect of Na-Alg and MAA on Swelling

Increasing the Na-Alg and MAA feed ratios in the formulations (SCH-4 to SCH-6 and SCH-7 to SCH-9) resulted in a more significant swelling of the developed hydrogels at pH 7.4. Na-Alg has a low cross-linking capability, thus resulting in more substantial swelling. By increasing the Na-Alg and MAA contents, the equilibrium swelling was increased from 535.18% to 743.19% and 421.25% to 478.56% in (SCH-4 to SCH-6) and (SCH-7-SCH-9), respectively, due to repulsion between –COO^−^ anions generated by the ionization of monomer and polymer at basic pH (9). Na-Alg has a lowly cross-linked structure, under which it has more swelling abilities. Higher Na-Alg concentration in the formulation thus expand more as compared to the formulations having lower concentrations. Afshar et al. (2020) have prepared ann Na-Alg/PVA-based hydrogel containing drug-loaded chitosan nanoparticles. The authors evaluated the equilibrium swelling% of the hydrogel and the effect of feed contents on its swelling%. Furthermore, they concluded that Na-Alg’s weak cross-linking tendencies presented better swelling behavior [25].

### 3.5. Effect of MBA on Swelling %

Higher MBA content in the formulations (SCH-10 to SCH-12) results in high cross-linking density, so the swelling was decreased from 550.70% to 311.65%. This reduction in swelling was attributed to the formation of a dense network that resists the penetration of swelling media, resulting in poor swelling. Similar findings were reported by Thakur and Arotiba (2018) in a study conducted on Na-Alg-co-AA hydrogels. They identified that the influence of MBA on the swelling ability follows a power law, i.e., Swelling capacity and MBA (cross-linker) content are inversely proportional [26]. The results for the DF loading (%) and equilibrium swelling (%) are presented in Figure 3.

### 3.6. Sol-Gel Fraction Analysis

**Figure 4:**
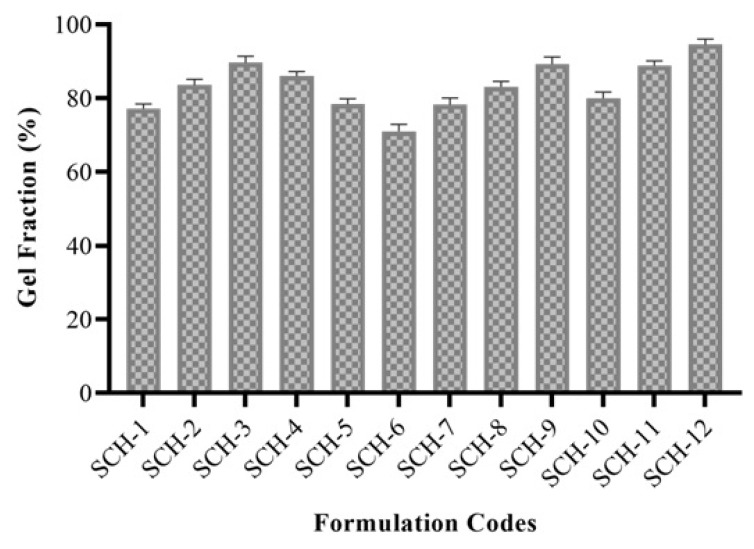
Gel fraction (%) of developed formulations (SCH-1 to SCH-12).

The impact of ratios of various feed contents on the gel fraction is shown in Figure 4. It is revealed that, by increasing the CP while keeping the concentrations of other feed components constant in formulations (SCH-1 to SCH-3), the gel fraction was increased from 77.10% to 89.66%. This increase in the gel fraction at higher CP concentrations may be attributed to the availability of more reactive sites for monomer polymerization [27,28]. The formulations developed with varying amounts of Na-Alg (SCH-4 to SCH-6) showed a decrease in gel fraction from 85.94 to 71.00% upon the rise of Na-Alg concentration. Low cross-linking tendency and high aqueous solubility are responsible for the decreased gel fraction [29]. Formulations developed with increasing concentrations of MAA (SCH-7 to SCH-9) and MBA (SCH-10 to SCH-12) showed higher gel fractions, from 78.25 to 89.25% and from 80.0 to 94.58%, respectively. The improved gel fraction at higher MAA and MBA contents confirmed the formation of more a compact hydrogel due to a high cross-linking density [30,31].

### 3.7. FTIR Spectrophotometric Analysis

**Figure 5:**
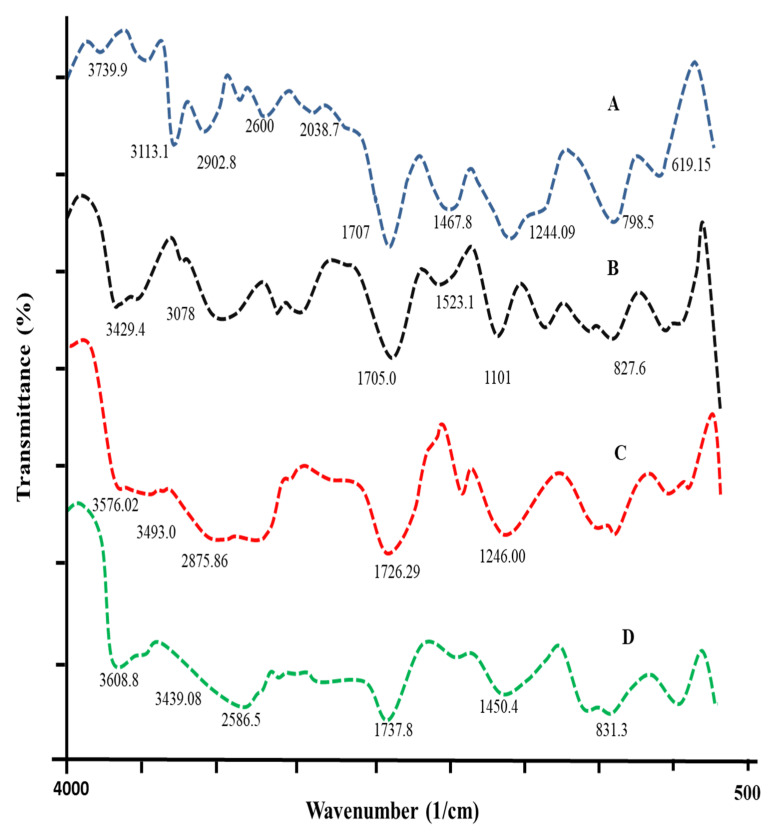
FTIR spectra of (**A**) Diloxanide Furoate, (**B**) Na-Alg, (**C**) Carbopol-934P, and (**D**) DF-loaded Na-Alg/CP –co-poly (methacrylate) hydrogels.

FTIR spectra of DF, Na-Alg, Carbopol-934P, MBA, and Na-Alg/CP co-poly (methacrylate) hydrogel were recorded and are shown in Figure 5. In Figure 5A, the C–H stretching peaks in the diloxanide furoate can be seen at 3113.11 cm^−1^. The ester group likely contributed to the C=O stretching seen at 1707.00 cm^−1^, whereas the ether group’s C–O–C stretching is observed at 1244.09 cm^−1^. At 798.53 and 619 cm^−1^, the C-Cl stretch reveals the existence of the halogen group [32].

In the IR spectrum of Na-Alg (Figure 5B), two bands appeared at 3429.43 cm^−1^ and 3078.39 cm^−1^ due to stretching movements of –OH and –CH_2_ groups, respectively. The peaks at 1523.16 cm^−1^ and 1705.07 cm^−1^ are related to the asymmetric and symmetric vibrations of the carboxylic groups. The peaks corresponding to the asymmetric stretching of the C–O–C groups appeared at 1101 cm^−1^, indicating glycosidic linkages of polysaccharides. A broader peak corresponding to the C–H deformation of mannuronic acid residue appeared at 827.46 cm^−1^ [33].

The FTIR spectrum of Carbopol 934P (Figure 5C) showed characteristic peaks at 3576.02 cm^−1^, 3493.09 cm^−1^, and 3342.60 cm^−1^ corresponding to –OH stretching movements. The peak that appeared at 2875.86 cm^−1^ was assigned to the stretching vibration of R–CH_2_ [34]. A sharp peak at 1726.29 cm^−1^ indicated a C=O stretching vibration, while a peak at 1246 cm^−1^ suggested a bending vibration of the –OH groups.

The FTIR spectrum of the developed Na-Alg/CP-co-poly(methacrylate) hydrogel (Figure 5D) indicated a broad shift of the characteristic peaks due to the –OH stretching vibrations at 3439.08 cm^−1^ and 3608.81 cm^−1^. A new peak appeared at 2586.54 cm^−1^ and was assigned to the stretching methylene cross-linker groups (MBA). A shift of the peaks corresponding to the C=O stretching occurred at 1737.86 cm^−1^. Our findings were similar to a study by Sarfraz et al. (2020) that evaluated and confirmed grafting in Carbopol-g-MAA-based nanogels [35].

### 3.8. Thermal Analysis

**Figure 6:**
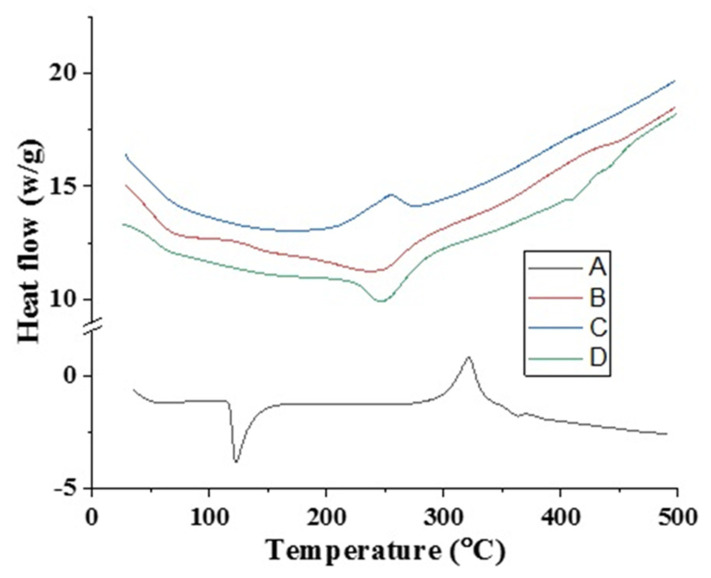
DSC thermogram of (**A**) DF, (**B**) Na-Alg, (**C**) CP-934P, (**D**) DF-loaded Na-Alg/CP-co-poly (MAA) hydrogel.

**Figure 7:**
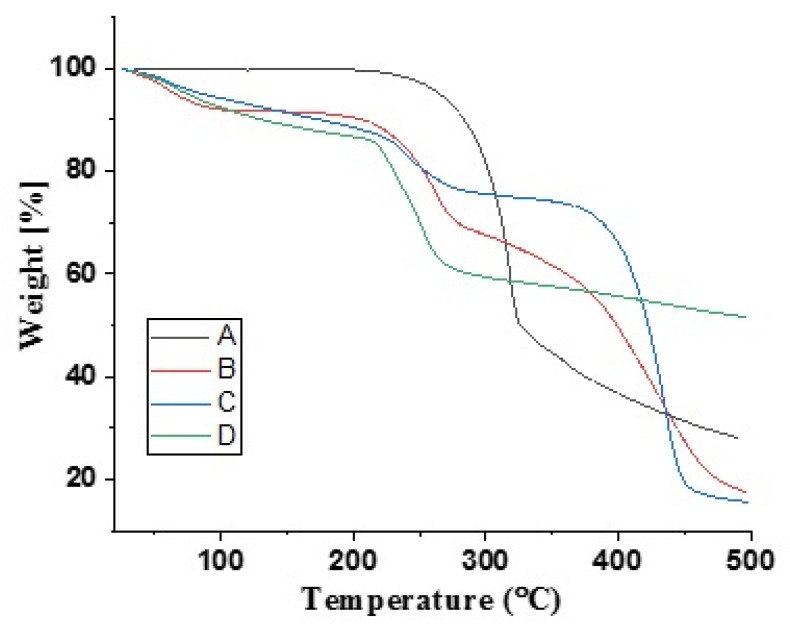
TGA thermograms of (**A**) DF, (**B**) Na-Alg, (**C**) CP-934P, (**D**) DF-loaded Na-Alg/CP-co-poly (methacrylate) hydrogel.

The DSC and TGA thermograms of pure DF, Na-Alg, CP-934P, and Na-Alg/CP-co-poly (methacrylate) hydrogels are shown in Figure 6 and Figure 7. The DSC thermogram of DF revealed its phase transition behavior through a sharp endothermic peak at around 115°C (Figure 6A). This peak corresponds to its melting point. Shukla et al. (2014), in a study conducted on Na-Alg microbeads loaded with DF, reported an endothermic peak corresponding to the melting point of DF at 115°C [36]. The DSC thermogram of Na-Alg showed an exothermic peak at 263.6°C, suggesting the thermal degradation of Na-Alg (Figure 6B). A similar peak at 220°C was reported by Xiao et al. (2000) in a study conducted to evaluate the thermal stability of blend films developed with a blend of Na-Alg and gelatin [37]. The DSC thermogram of CP showed two endothermic peaks at 68.2°C and 238°C (Figure 6C). The peak at 68.2 °C may correspond to the escape of the free fraction of water in the CP; however, the peak at 238°C is attributed to the formation of anhydrides in the polymeric chains. The same pattern was reported by Amrutkar et al. (2009) in their study conducted to evaluate the thermal parameters of developed matrix tablets of indomethacin for colon-targeted delivery [38]. The DSC thermogram of the developed Na-Alg/CP-poly(MAA) hydrogel (SCH-9) revealed an endothermic peak at 240°C (Figure 6D), which indicates the melting of the developed polymeric network.

Around 245°C to 250 °C, DF decomposes, as seen in the significant weight loss (%) on the TGA thermogram (Figure 7A). A loss of bound or free water in Na-Alg might explain why the TGA thermogram for pure Na-Alg revealed just a 14% weight loss up to 213.73°. Because of its hydrophilicity, it readily absorbs water from its surroundings, leading to a 37% reduction in mass after being exposed to very high temperatures (about 263.6 °C) (Figure 7B). Na-alginate depolymerization may explain the percentage weight loss seen at 488.25 °C.

The TGA thermography revealed a three-stage weight reduction for CP. The initial CP weight reduction of 8% might have been due to its losing moisture in the first step. During the second phase of weight loss, between 213 and 263 degrees Celsius, 15% of the weight was lost due to decarboxylation and the formation of unsaturated structures within polymer chains. After the third step, the CP began to depolymerize and break down, as shown by a 22.55 percent weight loss from 363 to 463 degrees Celsius (Figure 7C), until the whole polymer structure was paralyzed [39].

The TGA thermogram of the finished hydrogel revealed that the weight was lost in two distinct stages, at 220 degrees Celsius (12.61%) and 368 degrees Celsius (31.49%). At 500 degrees Celsius, the breakdown process began, leaving behind 38.78% of its original weight as residue (Figure 7D). This means the newly designed polymeric hydrogel is stable for longer and can withstand greater temperatures.

The TGA and DSC analysis results suggested that the developed hydrogel networks have improved thermal stability compared to their individual constituents.

### 3.9. XRD Analysis

**Figure 8:**
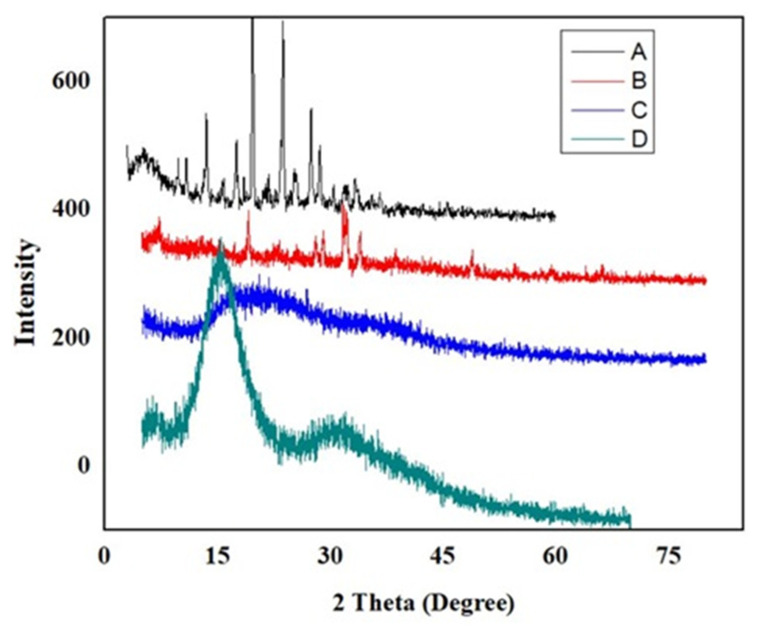
XRD diffractograms of (**A**) Diloxanide Furoate, (**B**) Na-Alg, (**C**) CP-934P, (**D**) Diloxanide Furoate loaded Na-Alg/CP-co-poly (methacrylate) hydrogel.

X-ray diffraction analysis was performed to evaluate the diffraction pattern and the effect of cross-linking on the crystallinity of DF. The pure DF diffractogram showed its characteristic peaks at 2*θ* = 13.5°, 17.5°, 19.6°, 23.7°, and 27.45°, indicating its crystalline nature. The Na-Alginate diffractogram suggested the crystallinity of Na-Alginate as exhibited by intense peaks at 7.2°, 19.08°, 28.1°, 29.08°, 31.68° and 34.04°, whereas CP 934P showed much broader and more diffused peaks of low intensity, which indicate its amorphous character. The loaded hydrogel (formulation SCHL-9) showed broader peaks of low intensity, suggesting the successful incorporation of DF into the polymeric network, as shown in Figure 8. The crystalline character of DF has been converted into an amorphous nature, as seen in the XRD diffractogram, in which all the prominent, apparent, and intense peaks have been turned into fused and less intense peaks.

### 3.10. EDX Studies

Energy dispersive X-ray spectroscopy (EDX) was carried out to evaluate the elemental composition and the weight% of each detected element in the developed unloaded Na-Alg/CP-co-poly(methacrylate) hydrogel and the DF-loaded Na-Alg/CP-co-poly(methacrylate) hydrogel.

**Figure 9:**
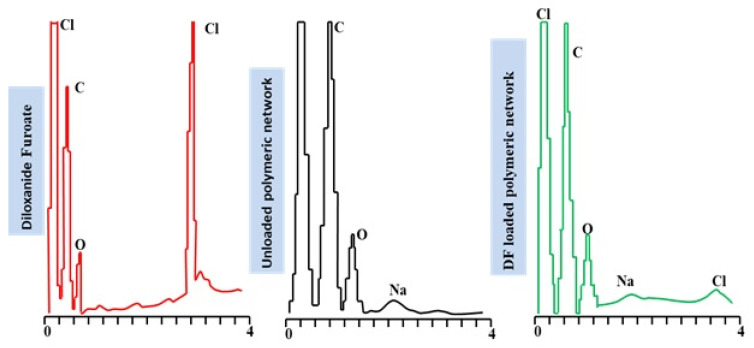
EDX spectra of Diloxanide Furoate, unloaded Na-Alg/CP-co-poly(methacrylate) hydrogel, DF-loaded Na-Alg/CP-co-poly(methacrylate) hydrogel.

The EDX emission spectrum of the unloaded hydrogel formulation SCH-9 indicated the presence of C, O, and Na as prominent peaks. In contrast, the loaded hydrogel spectrum revealed the presence of another Cl peak accompanied by a reduction of the O and Na peaks’ intensity. The Cl peak into the developed hydrogel suggested the uptake of the DF. Figure 9 and Table 2 present the prominent peaks along with the weight % and atomic % of each constituent element.

According to the quantitative elemental analysis, diloxanide furoate was found to have 68.20% carbon, 20.12% oxygen, and 11.67% chlorine. According to Table 2, the carbon, oxygen, and chlorine concentrations in unloaded hydrogels are 59.50%, 40.12%, and 0.38%, while they were 59.83%, 39.55%, 0.15%, and 0.46% in DF-loaded hydrogels.

### 3.11. SEM Analysis

**Figure 10:**
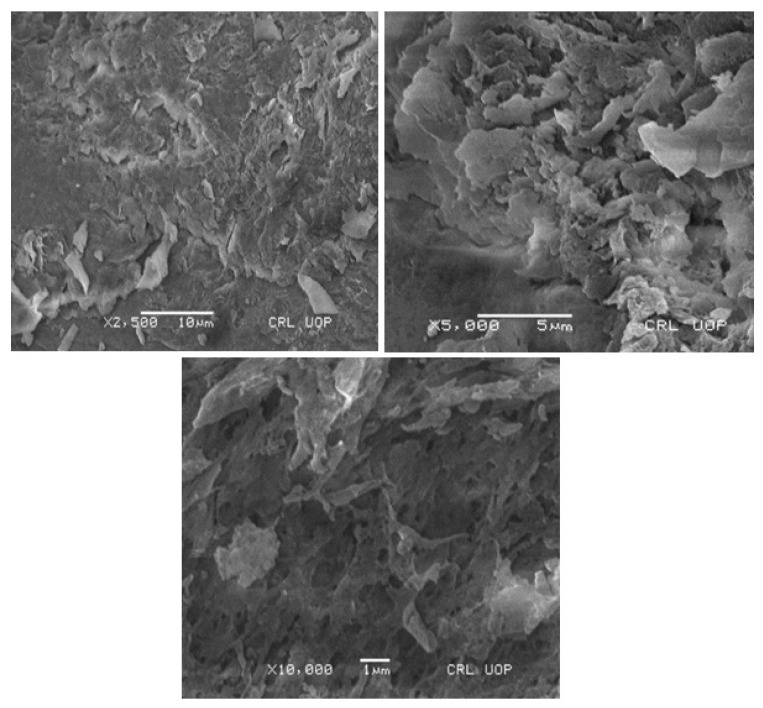
SEM micrographs of developed Na-Alg/CP co-poly(methacrylate) hydrogel.

A surface electron microscopy examination was used to examine the morphology of the hydrogels made from Na-Alg/CP-co-poly(methacrylate) (SCH-9). At 2500×, 5000×, and 10,000× magnifications, the surface morphology of the formed polymeric network was quite rough, as illustrated in Figure 10. Due to the porous surface structure, the pendant groups the polymer and the monomer provide may have an electrostatic interaction. Dimensional networking in the hydrogel causes open pores, which in turn causes optimum swelling, drug loading, and drug release [23,40].

### 3.12. In-Vitro Drug Release Studies

The in-vitro drug release testing was done on all 12 formulations at pH 1.2 and 7.4. There was a significant discrepancy between the medicine release at acidic pH of 1.2 and basic pH of 7.4. Minimal DF release (less than 10%) has been seen at pH 1.2 across all formulations (SCH-1 to SCH-12). Due to the acidic pH, the carboxylic groups remained unionized. Consequently, releasing or biological media had trouble penetrating the networks due to the lack of polymeric chain expansion, and the DF was poorly dissolved. In solution, carboxylic acid groups are ionized into carboxylate ions at a pH of 7.4. These ionic species stimulate repulsive forces between the polymeric chains, resulting in the formation of spaces. The integrated moiety dissolves as the dissolving medium goes inward. Since there is a concentration gradient, the dissolved DF travels away from the center of the growing hydrogel. The hydrogels’ release profiles were profoundly affected by the relative amounts of polymer, monomer, and cross-linker used in their production. At pH 7.4, DF release was reduced when the concentration of CP-934 P in formulations was increased (SCH-1 to SCH-3). Hydrogels with a tight structure, such as those produced by a high concentration of carbopol, may be responsible for the reduced drug release. Limited drug release occurs because solvent penetration into such a dense network increases viscosity and shrinks micropores. Bera et al. (2015) developed Carbopol-based microbeads with Mucoadhesive potentials to facilitate the delivery of the anti-diabetic medication Glipizide. The same research group also found that CP promotes adhesiveness and inhibits medication release [41].

**Figure 11:**
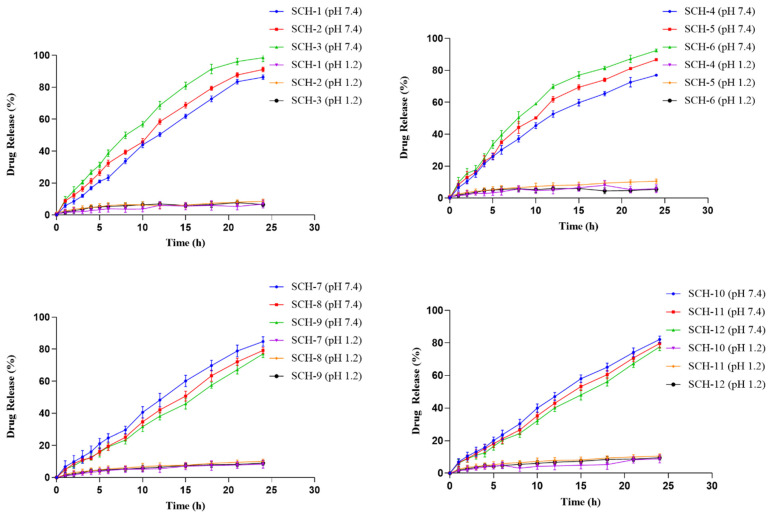
Effect of CP-934P, Na-Alg, MAA, and MBA on the DF release % from the developed hydrogel formulations (SCH-1 to SCH-12).

The Na-Alg and MAA levels in formulations (SCH-3 to SCH-6 and SCH-7 to SCH-9) enhanced the diloxanide furoate release from 77.0 to 91.77 percent and from 77.08 to 84.77 percent, respectively (Figure 11).

The increased swelling at higher Na-Alg and MAA concentrations is partly due to the massive number of pendant –COOH groups in the polymeric chains of these substances. –COOH group protonation and deprotonation significantly affect the release profile. The lack of repulsion between the pendent groups at acidic pH reduces the pore size, limiting drug release. This is because the protonation of COOH groups at an alkaline pH forms –COO– ions, which cause a significant release. Large pores formed due to electrostatic repulsion between the particles allow rapid medication release [13]. The carboxyl groups’ role in the pH-dependent release of loaded medication has also been studied elsewhere [42,43].

The DF release rate decreased from 81.63 to 67.85% when the MBA concentration in the synthesized formulations increased from SCH-10 to SCH-12. The enhanced cross-linking of the polymeric components with the increase in MBA concentration may account for this diminution. These results are consistent with the published literature [31,44].

Mathematical models were applied to investigate and explain the in-vitro release kinetics of DF from the loaded hydrogel in phosphate buffer using the first order, zero order Korsemeyer Pappas, and Higuchi model. The mathematical model that best matches the release data was determined by calculating the coefficient of determination, or “R^2^.” As can be shown in Table 3, the zero-order release kinetics model has a slightly higher “R^2^” than the first-order and Higuchi models for all formulations (SCH-1 to SCH-12).

This suggests that that the drug release from the gel network that occurs is controlled via pore diffusion, regardless of the drug concentration at the application site. The Korsmeyer Pappas model was applied to evaluate the drug release mechanism from the loaded hydrogels by using the release exponent “n”. The formulations SCH-3 to SCH-6 showed a value of n greater than 0.45 but less than 0.89, suggesting an anomalous (non-Fickian) diffusion of drug release. In contrast, all other formulations (SCH-1, SCH-2, and SCH-7 to SCH-12) represented super case II transport associated with values greater than 0.89. The case-II anomalous diffusion process is linked with controlled swelling.

### 3.13. Ex-Vivo Mucoadhesion

The assessment of the mucoadhesive force was done by increasing the weights on the right pan of the beam balance until the detachment of the two vial surfaces occurred.

**Figure 12:**
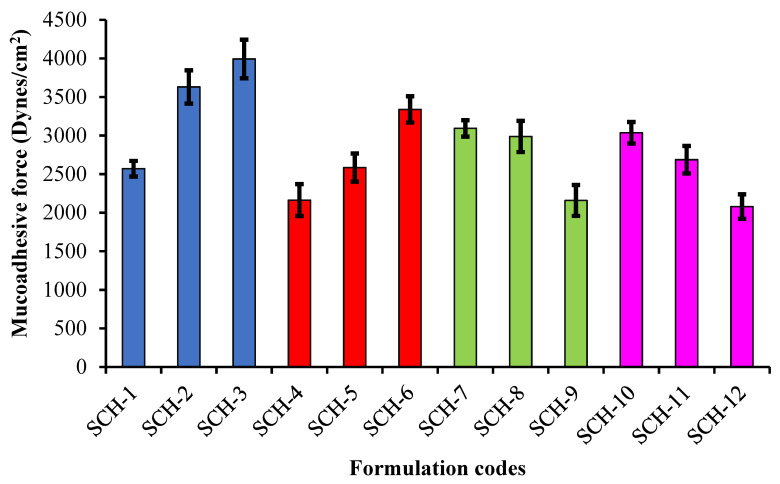
Comparison of the Mucoadhesive force (Dynes/cm^2^) of developed formulations (SCH-1 to SCH-12) (*p*-value > 0.05).

In the formulations SCH-1 to SCH-3 and SCH-4 to SCH-6, when increasing the concentration of CP-934P and Na-Alg, respectively, a rise in bioadhesion with the intestinal mucosa of rabbits was recorded. This may be because the prolonged contact of the hydrogel network in an aqueous medium resulted in the uncoiling of polymeric chains with subsequent penetration of these chains into the mucous. Several theories are quoted in the literature to explain the interaction of adhesive products with mucin, i.e., the electrostatic adsorption, wetting, diffusion, and fracture theories. The rinsing approach is adapted to determine mucoadhesive force [44]. A higher content of CP-934P and Na-Alg offers a more significant number of ionizable –COOH functional groups and many polymeric chains for interpenetration into the mucous membrane. Figure 12 represents the mucoadhesion force (dynes/cm^2^) for all the formulations. The hydrogen bridging between the –COOH groups and the sialic acid residues of mucous contributes to the adherence of the developed hydrogel to the mucous membrane. Formulations developed with higher contents of MAA and MBA showed a reduction in the mucoadhesion force, attributed to bigger cross-linking, making fewer functional groups available for interaction with the mucin. The findings align with the authors’ results, revealing a direct relationship between the amounts of Na-Alg and the mucoadhesive strength of the developed microparticles [45].

Incorporating a CP-934P, a synthetic polymer, into a hydrogel network renders high mechanical strength to the hydrogel due to its bioadhesive character. This inclusion also improves the toughness of the developed network, which can withstand the contractile forces of GIT [46].

### 3.14. Acute Oral Toxicity Studies

To determine the safety of the generated polymeric network, acute oral toxicity tests were performed. The rabbits were split into Group I (control) and Group II (treated group). Na-Alg/CP-co-poly(methacrylate) hydrogel-grounded particles with water assistance were delivered to the treatment group, whereas the control group received no treatment.

We observed the animals for a total of 14 days to see whether the treatment altered their feeding patterns, behavior, or overall health in any way. There was a lot of drooling, crying and itchy skin, and a stuffy nose. Redness, inflammation, and tears were not seen in the treated animals. There were no unexplained deaths or changes in the treated group’s water or food intake.

On day 14 of therapy, blood was collected from both groups for hematological and biochemical examination to determine whether or not the treatment impacted organ function. There was no discernible difference in the biochemical data between the treatment and control groups. Table 4 and Table 5 show the outcomes of the hematological analysis and other toxicity indicators.

Table 6 displays the results of hepatic and liver function tests, which revealed that the Na-Alg/CP-poly(MAA) hydrogels were well tolerated and did not cause any functional abnormalities.

On the 14th day after dosing, the animals from both groups were sacrificed, and their vital organs were removed to investigate any histo-pathological signs related to the treatment. The organs, such as the brain, liver, spleen, kidney, lungs, heart, and intestine, were preserved in formalin 10% solution and then examined microscopically for histopathology after being stained with H and E dyes. The gross morphology of the vital organs of the control and treated group is shown in Figure 11.

**Figure 13:**
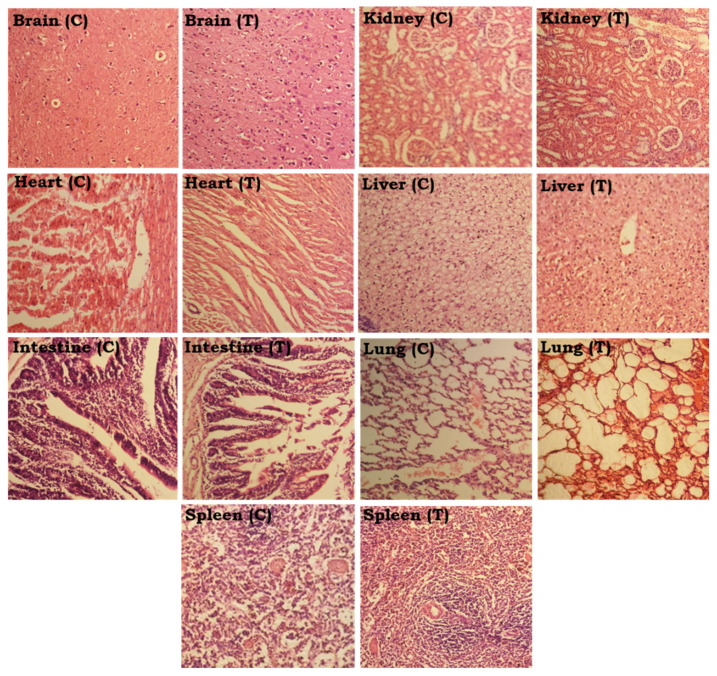
Images of the vital organs of the control group (C) and the Na-Alg/CP-poly(MAA) hydrogel-treated group (T) fourteen days after oral administration.

The brain showed the presence of excessive glial cells with minimal shrinkage of the neurons. Overall, no signs of damage were seen in the axonal neurons. The heart tissue histology suggested no localized hypoxia, damage, or cardiomegaly. Only a few cells showed signs of morphological artifacts in some areas. The lung tissues in the microscope showed signs of hemorrhage visualized in the spleen tissue with normal alveolar wall thickness. There was no sign of alveolar edema. The kidney tissue appeared normal with a normal glomerulus and bowman capsule. The shape and appearance of the spleen were fine on the whole, with healthy-looking red and white pulp zones. Splenomegaly was not seen. Slight hepatocyte ballooning, indicative of liver fibrosis, is seen in Figure 13 (T).

## 4. Conclusions

Hydrogels based on Na-Alg/CP-934P-co-poly(MAA) were developed using a free radical polymerization approach, revealing themselves as a smart and environmentally sensitive network enabling pH-driven regulated distribution of DF to the colon. Adding Na-Alg and Carbopol-934P increased the material’s biodegradability, Mucoadhesion, and mechanical strength. The manufactured network’s increased toughness makes it an excellent choice for withstanding the contractile forces of GIT.

## Figures and Tables

**Table 1:** Composition of Na-Alg/CP-co-poly (MAA) hydrogels (SCH-1 to SCH-12).

Codes	Carbopol 934P (g)	Na-Alg (g)	MAA (g)	MBA (g)	BPO (g)
SCH-1	0.15	0.25	6	0.15	0.15
SCH-2	0.30	0.25	6	0.15	0.15
SCH-3	0.45	0.25	6	0.15	0.15
SCH-4	0.15	0.4	6	0.15	0.15
SCH-5	0.15	0.55	6	0.15	0.15
SCH-6	0.15	0.7	6	0.15	0.15
SCH-7	0.15	0.7	9	0.15	0.15
SCH-8	0.15	0.7	12	0.15	0.15
SCH-9	0.15	0.7	15	0.15	0.15
SCH-10	0.15	0.7	12	0.25	0.15
SCH-11	0.15	0.7	12	0.35	0.15
SCH-12	0.15	0.7	12	0.45	0.15

**Table 2:** Elemental Composition of pure DF, unloaded, and DF-loaded networks.

Materials	Elements	Weight (%)	Atomic (%)
Pure Diloxanide Furoate	C O Cl	68.20 20.12 11.67	78.16 17.31 4.53
Developed Na-Alg/Carbopol-co-poly(methacrylate) hydrogels	C O Na	59.50 40.12 0.38	66.25 33.53 0.22
Diloxanide Furoate loaded Na-Alg/carbopol-co-poly(methacrylate) hydrogels	C O Na Cl	62.09 37.00 0.36 0.54	68.80 30.78 0.21 0.20

**Table 3:** Results of Kinetic modeling on release data (SCH-1 to SCH-12)**.**

Formulations	ZO	FO	Hig	KP	
	R^2^	R^2^	R^2^	n	R^2^
SCH-1	0.9961	0.9607	0.8513	0.900	0.9967
SCH-2	0.9864	0.9683	0.8853	0.916	0.9952
SCH-3	0.9721	0.9658	0.9180	0.729	0.9896
SCH-4	0.9847	0.9689	0.8933	0.799	0.9955
SCH-5	0.9948	0.9678	0.9042	0.774	0.9954
SCH-6	0.9948	0.9664	0.9232	0.704	0.9839
SCH-7	0.9959	0.9611	0.8442	0.913	0.9951
SCH-8	0.9955	0.9565	0.8279	0.956	0.9955
SCH-9	0.9969	0.9526	0.8110	0.907	0.9978
SCH-10	0.9937	0.9686	0.8436	0.918	0.9966
SCH-11	0.9973	0.9611	0.8215	0.978	0.9980
SCH-12	0.9976	0.9547	0.8056	0.923	0.9979

**Table 4:** Parameters for assessing acute oral toxicity.

Observations	Symptoms	Control	Treated
Body Weight (g)			
1st Day 7th Day 14th Day		1.355 ± 0.02 1.290 ± 0.03 1.309 ± 0.02	1.373 ± 0.01 1.369 ± 0.21 1.372 ± 0.40
Water Intake (mL)			
1st Day 7th Day 14th Day		156.76 ± 1.54 149.19 ± 1.77 171.98 ± 2.41	160.9 ± 0.32 172.53 ± 0.04 173.69 ± 0.95
Food Intake (g)			
1st Day 7th Day 14th Day		70.96 ± 0.82 77.22 ± 0.69 73.67 ± 2.09	56.11 ± 3.49 78.68 ± 1.12 69.93 ± 3.04
Dermal Irritation	Ulceration, Hair fall	No	No
Ocular toxicity	Redness, Lacrimation	No	No
Motor activity	Hyperactivity, Restlessness	Absent	Absent
Mortality	Death	No	No

Mean ± S.D. (SD = Standard Deviation).

**Table 5:** Hematological examination of the blood of rabbits.

Parameters	Group 1 (Control)	Group II (Treated)
Hemoglobin (g/dL) (10–15 g/dL)	12.3 ± 0.02	14.1 ± 0.23
White blood cells (×10^3^ /µL)	6.6 ± 0.09	5.96 ± 0.01
Red blood cells (3.8–7.9 × 10^6^ /µL)	6.55 ± 0.03	5.79 ± 0.42
Platelets (×10^3^/ µL)	806 ± 0.13	793 ± 0.04
Monocytes (%)	3.71 ± 0.08	4.64 ± 0.13
Neutrophils (%)	50.43 ± 0.01	53.51 ± 0.20
Lymphocytes (30–70%)	43.4 ± 0.00	59.1 ± 0.08
Mean corpuscular volume (50–75 fL)	60.2 ± 0.06	56.64 ± 0.13
Mean corpuscular hemoglobin (pg/cell)	18.8 ± 0.16	16.32 ± 0.08
MCHC (27–34 g/dL)	31.2 ± 0.12	29.58 ± 0.09

Mean ± S.D. (SD = Standard Deviation).

**Table 6:** Biochemical analysis.

Analysis	Group I (Control)	Group II (Treated)
ALT (IU/L)	82 ± 0.1	85 ± 0.01
AST (IU/L)	45 ± 0.04	48 ± 0.02
Urea (mmol/L)	26 ± 0.02	36 ± 0.10
Creatinine (dg/dL)	1.09 ± 0.24	1.00 ± 0.01
BUN (mg/dL)	12 ± 0.01	17 ± 0.05
Cholesterol (mg/dL)	37.70 ± 0.25	37.95 ± 0.06
Triglycerides (mg/dL)	112.10 ± 0.14	129.34 ± 0.10
LDL (mg/dL)	12 ± 0.50	11.17 ± 0.11
VLDL (mg/dL)	22 ± 0.07	23 ± 0.34
Cholesterol /HDL	2.1 ± 0.26	2.4 ± 0.20

Mean ± S.D. (SD = Standard Deviation).

## Data Availability

Research data can be provided by corresponding authors on reasonable request.
